# Long Term Outcomes After Renal Revascularization for Atherosclerotic Renovascular Disease in the ASTRAL Trial

**DOI:** 10.1161/CIRCINTERVENTIONS.123.013979

**Published:** 2024-08-15

**Authors:** Hannah O’Keeffe, Darren Green, Aine de Bhailis, Rajkumar Chinnadurai, Keith Wheatley, Jonathan Moss, Philip A. Kalra

**Affiliations:** Renal Medicine, Salford Royal Hospital, United Kingdom (H.O.K., D.G., A.d.B., R.C., P.A.K.).; Faculty of Biology, Medicine and Health, University of Manchester, United Kingdom (H.O.K., D.G., R.C., P.A.K.).; Cancer Trials Research Unit, Birmingham, United Kingdom (K.W.).; School of Cardiovascular and Metabolic Health, University of Glasgow, United Kingdom (J.M.).

**Keywords:** atherosclerosis, blood pressure, follow-up studies, kidney, renal artery

## Abstract

**BACKGROUND:**

The ASTRAL trial (Angioplasty and Stenting for Renal Artery Lesions) recruited 806 patients between 2000 and 2007. Patients with atherosclerotic renal artery stenosis (RAS) and clinician uncertainty about the benefit of revascularization were randomized 1:1 to medical therapy with or without renal artery stenting. The initial results were presented in 2009 at a median 33.6-month follow-up, with no benefit of revascularization on renal or cardiovascular outcomes. Surviving patients remained under follow-up until the end of 2013, and the long-term results are presented in this study.

**METHODS:**

Data were analyzed to assess whether there was a later impact of revascularization on renal function, cardiovascular events, and survival, including a composite outcome of renal and cardiovascular outcomes and death (as in the CORAL trial [Cardiovascular Outcomes in Renal Atherosclerotic Lesions]). Prespecified subgroup analyses included different categories of renal function, rapid deterioration in kidney function, and degree of RAS. Post hoc analyses of patients with severe RAS (bilateral 70% or >70% in a solitary kidney), those with or without proteinuria, and a per-protocol analysis were performed.

**RESULTS:**

The mean age of the entry population was 70.5 years, the mean estimated glomerular filtration rate was 40 mL/min/1.73 m^2^, the mean RAS was 76%, and the mean blood pressure was 150/76 mm Hg; 83% of the revascularization group underwent attempted stenting. The median follow-up was 56.4 months, with 108 patients lost to follow-up. By the end of follow-up, 50% of the evaluable population had died, 18% had suffered a first renal event, and 40% had suffered a first cardiovascular event. No statistical difference was observed for any outcome in the intention-to-treat and per-protocol analyses.

**CONCLUSIONS:**

The long-term follow-up of the ASTRAL trial showed no overall benefit of renal revascularization to renal and cardiovascular outcomes. It has been highlighted that a proportion of the population had lower-risk RAS, and there is likely to be merit in further study in a higher-risk population.

**REGISTRATION:**

URL: https://www.isrctn.com; Unique identifier: ISRCTN59586944.

**WHAT IS KNOWN**

- Renal artery stenosis is a relatively common condition associated with atherosclerosis and hypertension.
- The ASTRAL study (Angioplasty and Stenting for Renal Artery Lesions) initially reported in 2009, with 806 patients randomized 1:1 to medical therapy or revascularization, and found no significant benefit from revascularization.

**WHAT THE STUDY ADDS**

- This article now presents the longest follow-up of any atherosclerotic renovascular disease trial, with surviving patients from the ASTRAL study followed for a median of 56.4 months.
- No difference was detected in any outcomes by intention-to-treat analysis.
- Revascularization is still performed in clinical practice on an individualized basis for those patients at the highest clinical risk (severe hypertension, heart failure, and rapidly progressive renal function), but after the results of previous randomized control trials, many patients with severe renal artery stenosis are likely to be consigned to conservative care. Further study of these subpopulations would be valuable.

Atherosclerotic renovascular disease is a common condition associated with generalized atheroma and aging that frequently leads to hypertension, chronic kidney disease (CKD), and heart failure.^1^ Historically, many patients with renal artery stenosis (RAS) have been treated with revascularization, with either primary or secondary (after angioplasty) stent placement. Several small and 2 large randomized controlled trials (RCTs) have sought to establish whether revascularization leads to clinical benefit in these patients.^2–6^ The ASTRAL trial (Angioplasty and Stenting for Renal Artery Lesions) included 806 patients with atherosclerotic RAS and reported no improvement in the primary outcome of renal function change over time in the stent plus medical therapy group compared with medical therapy alone.^5^ The CORAL trial (Cardiovascular Outcomes in Renal Atherosclerotic Lesions) had tighter inclusion criteria, with core radiological endorsement to ensure RAS lesions were physiologically significant, but it too showed no benefit of stenting plus medical therapy (statin, antiplatelets, and angiotensin receptor blocker) compared with medical therapy alone in the composite primary outcome of mortality and major cardiovascular and renal outcomes in the 947 randomized patients.^6^

Although the RCTs have concluded the absence of benefit from stenting, the trials had limitations, notably that the enrolled populations only included a minority of patients with high-risk clinical presentations (very severe hypertension, rapidly declining renal function, heart failure, and highly significant RAS), questioning the generalizability of the results to clinical practice.^5,6^ Additionally, both ASTRAL and CORAL did not enroll some patients who may have benefited from intervention at the preference of their treating physicians.^5,6^ In practice, many high-risk patients (those with severe hypertension, heart failure, or rapidly deteriorating kidney function) with high-grade RAS continue to undergo revascularization. The duration of follow-up at the point of reporting the outcomes of the 2 largest RCTs was relatively short, with a median follow-up of 34 months in ASTRAL and a median follow-up of 43 months in CORAL.^5,6^ As RAS lesions are known to progress even with the best available vasculo-protective therapy, with the probability of worsening renal ischemia, renal atrophy, progressive CKD, and impact on the wider vasculature, it would be an oversight to ignore the longer-term outcomes of the patients enrolled in the RCT.

ASTRAL enrolled 806 participants between 2000 and 2007, and the initial report included data after the last enrolled patient had received a 12-month follow-up in 2008. However, the trial design allowed for follow-up to be continued until the end of 2013 in patients who were surviving at the time of the first database lock (November 2008). This study reports the long-term outcomes of ASTRAL patients as specified in the original design of ASTRAL, including most prespecified subgroup analyses and additional analyses, including application of the CORAL composite primary outcome and key subgroups of very severe RAS and low-level proteinuria.

## METHODS

This was an analysis of long-term follow-up data from patients enrolled in the ASTRAL trial. Full details of the ASTRAL trial methods were described in the initial publication that included data from all randomized participants out to a minimum 12-month follow-up and a median of 33.6 months.^5^ The trial was approved by the West Midlands United Kingdom Multicenter Research Ethics Committee and the ethics committee at each participating study center. All patients provided written, informed consent. The data that support the findings of this study can be made available upon reasonable request to the authors.

In brief, patients with RAS confirmed by either computerized tomography, magnetic resonance, or intra-arterial imaging, and who were considered by their managing clinician to be potentially suitable for revascularization, were randomized to receive either standard medical therapy alone (determined by local policy but generally including statins and antiplatelets) or standard medical therapy with revascularization. The revascularization strategy was determined by local policy but invariably involved stent placement. The degree of RAS was determined by interventional radiologists in the individual trial centers, and a limitation was that there was no radiology core laboratory adjudication. Surviving subjects were followed up until December 31, 2013, so that the maximum possible follow-up was 13 years after enrollment. Data analysis had been delayed due to institutional governance processes that delayed obtaining the complete data set from the coordinating trial center, and further delays occurred due to resource limitations and commitments during the COVID-19 pandemic.

Participating sites collected data for each subject on the anniversary of their enrollment date on an annual basis beyond 2008, completing annual follow-up forms returned to the trial center. Key data returns were serum creatinine and clinic blood pressure (BP) closest to the annual anniversary date, need for renal revascularization (either cross-over from the medical care arm or repeat procedure in the revascularized arm), cardiovascular events (CVEs; myocardial infarction, stroke, death from cardiovascular causes, hospitalization for angina, fluid overload or cardiac failure, coronary artery revascularization, or peripheral arterial procedure), significant renal events (acute kidney injury, initiation of dialysis, renal transplantation, nephrectomy, or death from renal failure), and death, with cause of death noted when available.

The primary ASTRAL outcome was a change in renal function over time. For the current analysis, the reciprocal of creatinine used in the ASTRAL trial was replaced by an estimated glomerular filtration rate (eGFR) calculation using the CKD Epidemiology Collaboration equation. eGFR calculations were made retrospectively, and ethnicity adjustment was not included. This methodological adjustment reflects changes in clinical practice during the interval since ASTRAL was conceived, as the initial trial design predated widespread use of eGFR. The secondary outcomes included time to the first major renal event, the first CVE, and death, and were unchanged from the first trial report. A further secondary outcome was introduced for this analysis to mirror the composite end point of the CORAL trial; this was defined as time to the first of any renal, cardiovascular, or mortality events with the addition of time to >30% decline in renal function from enrollment (measured using the slope of eGFR over time).^6^ Follow-up was from trial enrollment until death, the most recent clinical review, or December 31, 2013. Subgroup analyses were completed as per the original trial design with the exception of kidney length. These included grouping by serum creatinine, eGFR, degree of RAS, and previous rapid progression of renal impairment (defined as an increase in serum creatinine >100 umol/L or >20% during the previous 1-year period), and post hoc analysis of severe RAS (>70% RAS either bilaterally or affecting a solitary functioning kidney) was also reported. An additional analysis according to the presence or absence of significant albuminuria (urinary albumin:creatinine ratio, stratified above or below 30 mg/mmol), estimated from overall measurements of proteinuria, was included in this study in recognition of an albuminuria subgroup analysis in the CORAL trial.^7,8^ As proteinuria in the ASTRAL patients was measured by urinary protein creatinine ratio or 24-hour total proteinuria, with urinary albumin:creatinine ratio derived from this, it was not possible to reliably use the CORAL urinary albumin:creatinine ratio cut-off, and the higher level of 30 mg/mmol was selected.^8^ The definitions and thresholds used in defining renal function subgroups were updated to eGFR-based CKD stages (using CKD Epidemiology Collaboration).

The annual change in eGFR was calculated for each patient after the end of the original trial period, and comparisons were made between revascularization and medical therapy at each yearly interval. Absolute values of eGFR at each annual time point were also the compared using *t* test. The same method was used for comparison of BP results. In cases of missing values for individual patients, the expected eGFR was imputed after calculation based on the eGFR slope before and after this time point, presuming a linear trend. This method of imputation was not applicable to the systolic BP outcome, as linear trends could not be presumed for BP.

For time-to-event outcomes, multivariate Cox proportional hazard models were constructed to compare randomized therapies from the time of enrollment into the trial, with a hazard ratio <1.0 indicating a benefit of revascularization. Covariates chosen a priori for inclusion in the model were baseline creatinine, age, and sex. These were selected to adjust for the potential confounding effect of the components of eGFR on the primary ASTRAL aim of detecting a change in renal function between therapies. Analyses were performed for the whole trial population and each of the subgroups as defined above. Additional per-protocol analyses were also performed. Log-rank test was used to assess the significance of the survival difference between the groups in the Kaplan-Meier charts. Throughout the analyses, a 2-sided alpha level was set at 0.05 for statistical significance. Analyses were performed using SPSS software, version 26 (IBM, New York). The imbalance of the baseline covariates in patients who were lost to follow-up was assessed by standardized mean difference calculations, and competing risk analyses were conducted using “tableone” and “cmprsk” packages in R software, version 4.3.1.

## RESULTS

**Figure 1.**
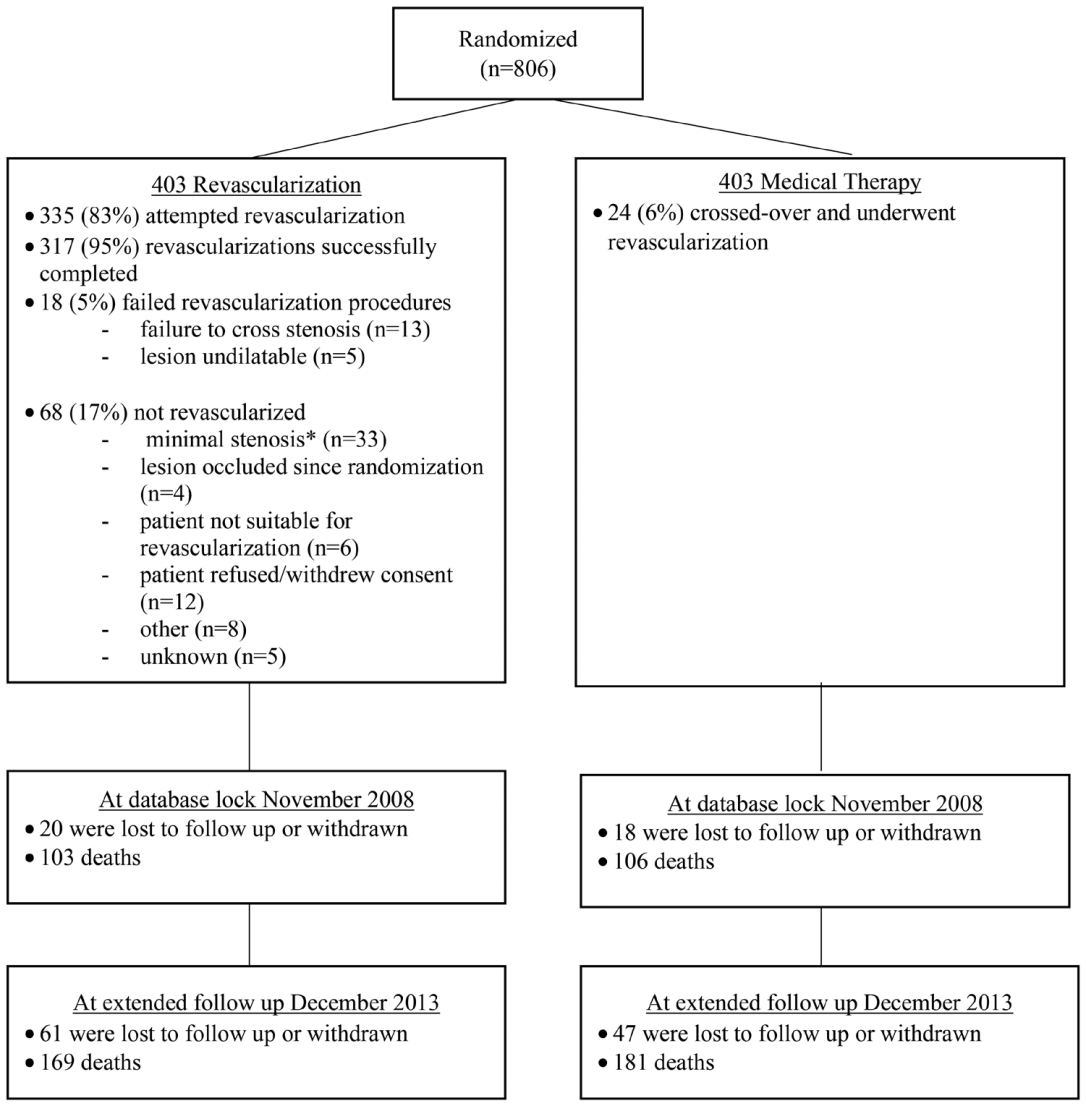
**CONSORT (Consolidating Standards of Reporting Trials) diagram for ASTRAL patient recruitment and follow-up.** ASTRAL indicates Angioplasty and Stenting for Renal Artery Lesions. *These patients were identified as having minimal stenosis post randomization determined by “on-the-table” intraarterial angiography.

The initial results of the ASTRAL trial, which enrolled 806 patients from 2000 to 2007, were reported in 2009, with surviving patients remaining under follow-up until December 31, 2013 (see CONSORT [Consolidating Standards of Reporting Trials] diagram in Figure 1). As of the end of 2013, median follow-up was 56.4 (interquartile range, 28.7–82.7) months compared with a median of 33.6 months in the 2009 publication. By the end of follow-up, 108 participants (13%) were lost to follow-up or had withdrawn from the study: 61 from the revascularization arm and 47 from the medical arm. There were no significant differences in the baseline covariates between the treatment arms in the lost-to-follow-up/withdrawn consent group (Table 1), and a competing risk model incorporating lost-to-follow-up as a censoring event did not show any significant difference in any of the outcomes (Table S1).

**Table 1.**
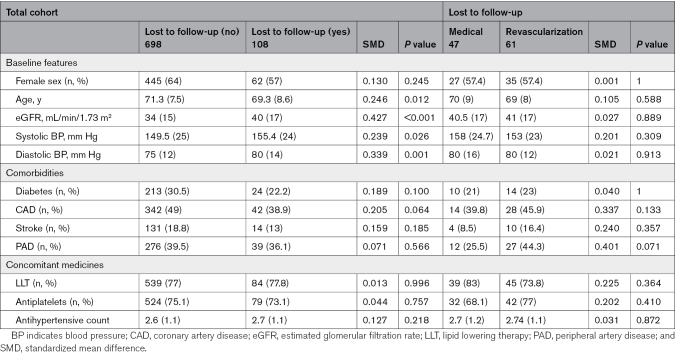
Assessment of Conditional Independent Censoring due to Patients Who Were Lost to Follow-Up

Table 2 shows the baseline data of the entire trial population and also that of key subgroups. The mean age of the participants at enrollment was 71 (SD, 7.6) years, the mean eGFR was 40 (range, 5.4–124.5) mL/min/1.73 m^2^, the mean RAS was estimated at 76% (range, 20%–100%), and the mean BP was 150/76 (SD, 25/12.4) mm Hg. Notably, 83% (335/403) of the patients randomized to receive revascularization underwent attempted stenting; the remaining 17% (68/403) of the revascularization group were either identified as having insignificant RAS (too minimal to merit revascularization) or fully occluded at the time of the planned procedure, or the patient had withdrawn consent.

**Table 2.**
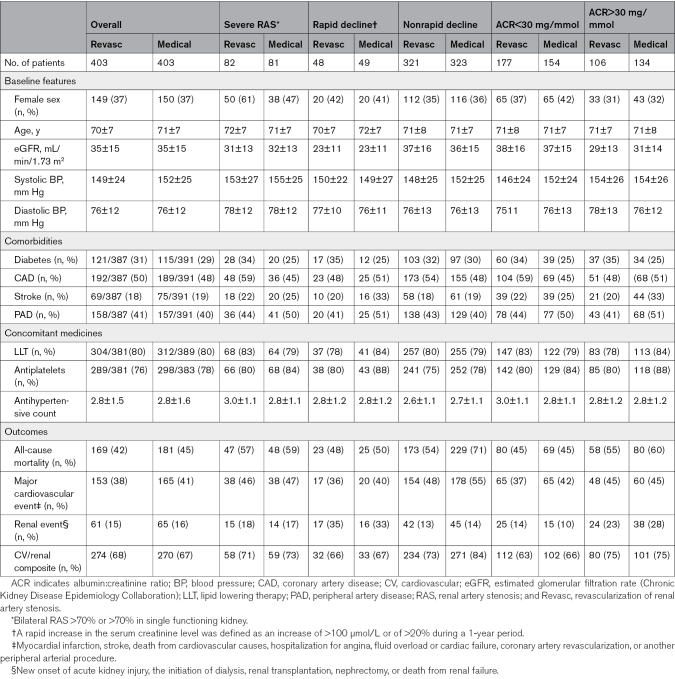
Baseline Characteristics, Comorbid Features, and Outcomes of Long-Term Follow-Up by Subgroup

Renal events occurred in 18% (126), including 14% (95) who required renal replacement therapy (RRT), and 46% (318) had suffered a first CVE. In ASTRAL, the original primary outcome was change in renal function, and prespecified secondary end points were BP, time to the first renal event, time to the first major CVE, and mortality. All of these prespecified end points were analyzed, and no differences were found. No difference was observed for any outcome in the intention-to-treat or per-protocol analyses, either in the whole population or the prespecified subgroups (see Table 3 and Figure S1). In an intention-to-treat analysis of the severe RAS subgroup of 163 patients (RAS >70% in both kidneys or in a single functioning kidney), revascularization was associated with a hazard ratio (HR) of 0.74 (95% CI, 0.54–1.01; *P*=0.062) for the composite renal and cardiovascular outcome (as used in CORAL) and a HR of 0.70 (95% CI, 0.49–1.0; *P*=0.051) for death (Table 4).

**Table 3.**
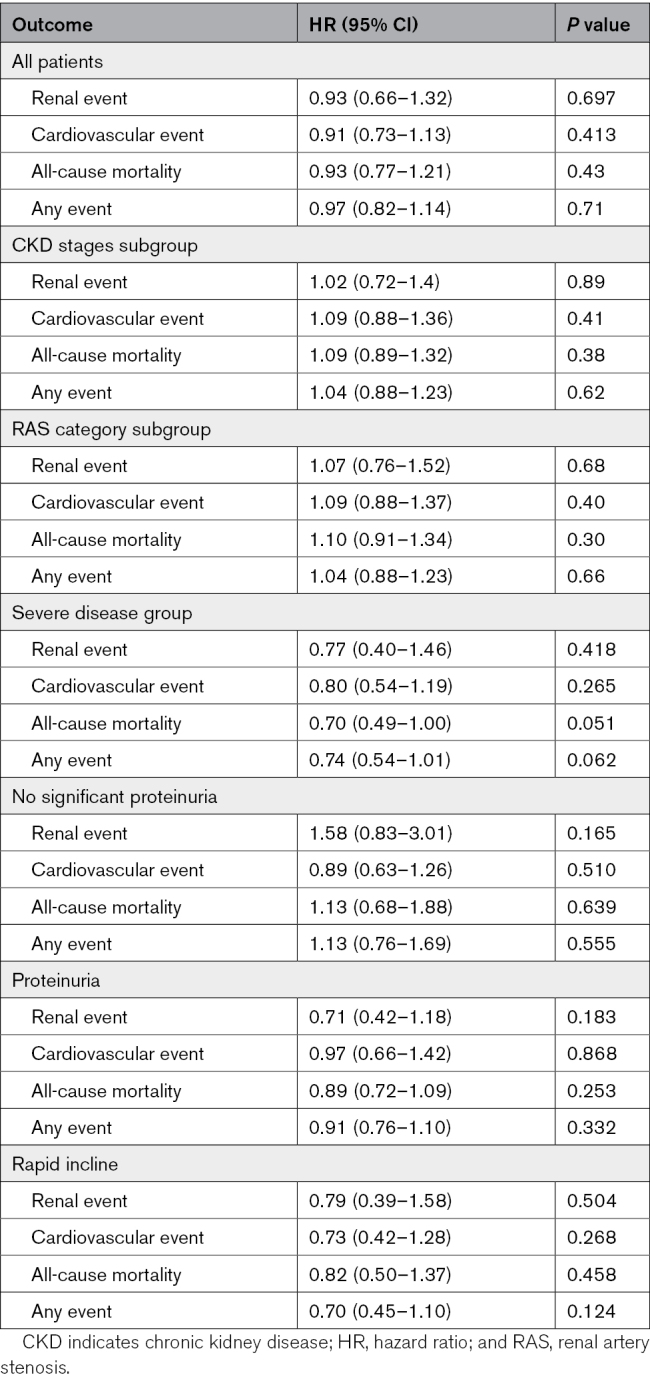
Multivariate Cox Proportional Hazard Model Showing Hazard Ratio for Outcomes in Revascularized Patients Compared With Medical Therapy as Reference Group (Adjusted for Age, Creatinine, and Sex at Baseline)

**Table 4.**
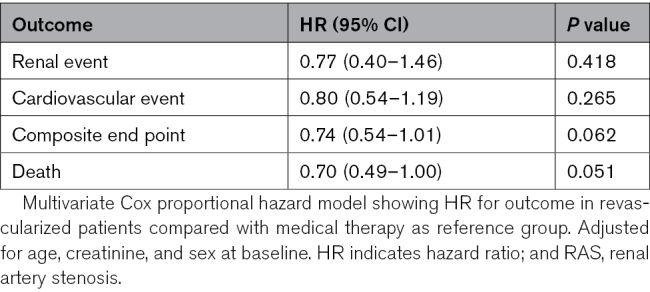
Outcomes for Patients With Severe RAS (Bilateral Stenosis >70% or >70% in a Single Functioning Kidney)

The median follow-up of patients in the revascularization group was 58.6 months, and 56.2 months in the medical group. At the time of analysis, 169 patients (42%) and 181 patients (45%) had died in these respective groups (*P*=0.727), whereas 61 (15.1%) and 47 (11.6%) had been lost to follow-up or withdrawn (*P*=0.147).

At the end of follow-up, the mean eGFR in the revascularization group was 38.6 versus 36.5 mL/min in the medical group (*P*=0.381).

### Renal and CVEs and Mortality

**Figure 2.**
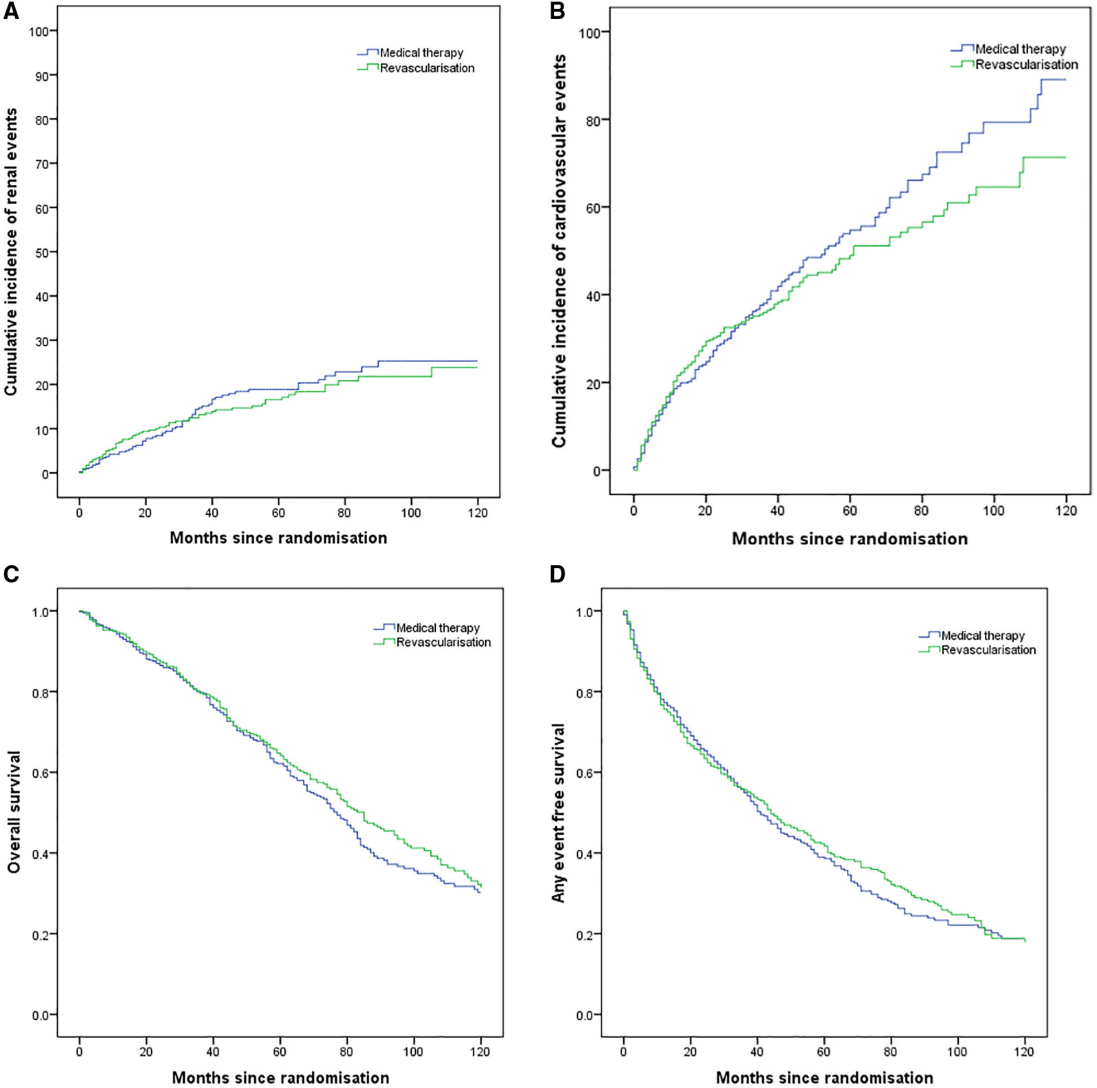
**Kaplan-Meier curves for secondary end points. A**, Time to first renal event (*P*=0.684), (**B**) time to first cardiovascular event (*P*=0.371), (**C**) overall survival (*P*=0.344), and (**D**) any event-free survival (CORAL outcome; *P*=0.710). CORAL indicates Cardiovascular Outcomes in Renal Atherosclerotic Lesions.

A total of 126 (18%) renal events were reported, with 61 (17.8%) in the revascularized group and 65 (17.9%) in the medical group. There was no significant difference in time to a renal event between the 2 groups (HR in revascularized group, 0.78 [95% CI, 0.53–1.15]; *P*=0.202; Figure 2A). In all, 43 (10.7%) patients in the revascularized group versus 50 (12.4%) in the medical group started dialysis; 2 patients in the revascularized group received a kidney transplant. The annual rate of progression to RRT was low at 1.29% in the medical group compared with 1.25% in the revascularization group. The majority of RRT events occurred early, with 4.2% (17) of the medical group requiring RRT in the first year versus 6.2% (25) in the revascularization group, and 1 or no individuals in either arm progressing to RRT annually after year 8.

A total of 609 CVEs were reported with 297 events in the revascularized group compared with 312 events in the medical therapy group. CVEs occurred at similar rates in the 2 groups (HR in the revascularized group, 0.98 [95% CI, 0.77–1.24]; *P*=0.864), and there was no difference in the time to the first CVE (*P*=0.387; Figure 2B).

Analysis similar to the CORAL composite primary end point, using a composite of time to the first renal, cardiovascular, or mortality events or time to >30% decline in renal function (by eGFR) from enrollment, also did not differ between the 2 groups (HR in the revascularized group, 0.98 [95% CI, 0.82–1.17]; *P*=0.777; Figure 2C).

There was no difference in overall survival (HR in the revascularized group 0.92, [95% CI, 0.75–1.12]; *P*=0.391) with a total of 350 deaths among those remaining in the trial, 169 of which were in the revascularized group and 181 in the medical group (Figure 2D). 5-year survival was 65.5% (264/403) in the medical group and 66.8% (269/403) in the intervention group, dropping to 47.4% (191/403) and 50.4% (203/403), respectively, at 10 years. The annual mortality rate over the entire follow-up period was 4.7% in the medical arm and 4.6% in the revascularization arm.

### Subgroup Analyses

No difference was observed between revascularization or medical therapy in any of the prespecified subgroups (according to serum creatinine, eGFR, degree of RAS, or previous rate of progression of renal impairment) in this long-term follow-up. However, in the intervening years since the original trial design, there have been significant changes in clinical practice and trial design. The clinically relevant subgroups are now felt to be severe RAS (bilateral >70% or >70% in a single functioning kidney), prior rapid declining kidney function, and the presence or absence of albuminuria. These subgroups and their outcomes are summarized across the revascularization and medical arms in Table 1. No statistical differences were found between revascularization and medical therapy in any of these subgroups.

**Figure 3.**
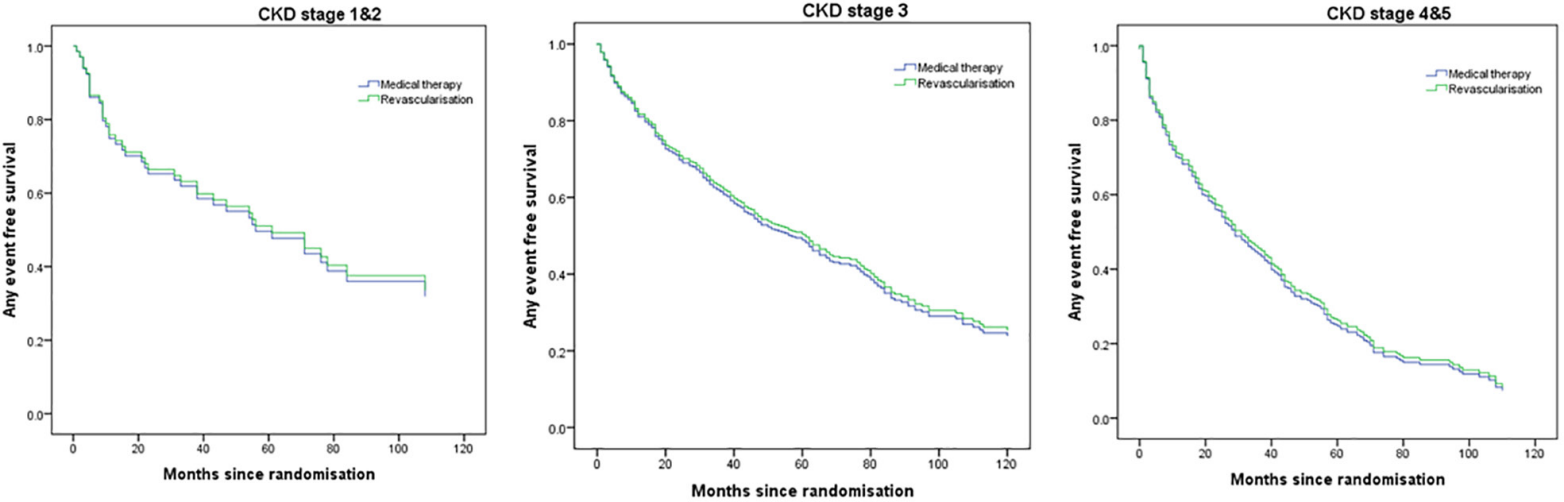
**Composite outcome (renal, cardiovascular, and mortality) event-free survival across CKD stages.** CKD indicates chronic kidney disease.

In the post hoc analysis of the severe RAS subgroup (bilateral stenosis >70% or stenosis >70% in a single functioning kidney), the HR for the CORAL composite end point was 0.74 (*P*=0.062) and for mortality 0.70 (*P*=0.051; Table 4). No difference was seen across the stages of CKD for any of the outcomes, including the composite outcome (Figure 3).

### Per-Protocol Analysis

Stenting was attempted in 335 and successful in 317 participants in the revascularization group. Twenty-four of those randomized to the medical group subsequently underwent revascularization. Thus, the per-protocol analysis included 317 in the revascularization arm and 379 in the medical arm. Overall, there was no significant difference in renal events, CVEs, the composite (CORAL) end point, or death when analyzed on a per-protocol basis (see Table 5).

**Table 5.**
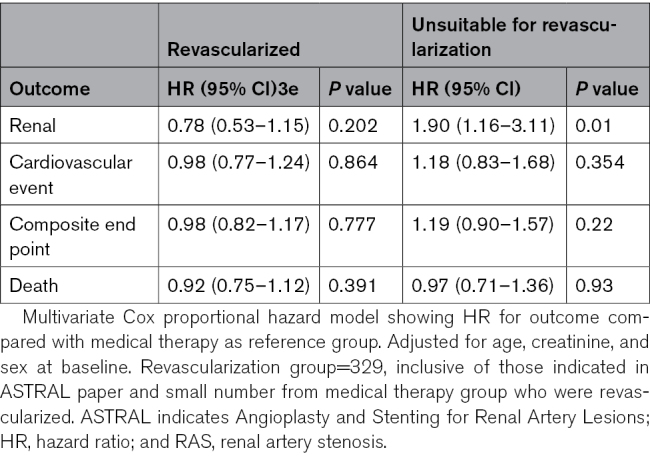
Per-Protocol Analysis

## DISCUSSION

The ASTRAL trial was designed to assess whether revascularization in addition to medical therapy for atherosclerotic RAS resulted in improved outcomes compared with medical therapy alone. The initial study report did not demonstrate any benefit of revascularization, and there were associated risks of intervention.^5^ The CORAL trial, published after ASTRAL and including a wider composite end point, also failed to demonstrate a benefit of revascularization in atherosclerotic RAS,^6^ although both RCTs had limitations regarding the randomized populations; in particular, the ASTRAL population comprised patients where the managing clinician felt there was uncertainty about the benefit of revascularization. This data set now presents the longest follow-up of any atherosclerotic renovascular disease RCT, which is of value given the high-risk nature of this population and hence the demonstration of the end points of mortality and cardiovascular and renal outcomes over time. The follow-up data showed no overall benefit of renal revascularization to renal and CV outcomes, although a trend towards significance was observed in both the composite CORAL end point and mortality in the severe RAS subgroup, albeit this was a post hoc analysis.

A post hoc analysis of the CORAL population found a significant difference in event-free survival in patients with an ACR ≤22.5 mg/g (equivalent to ≤2.83 mg/mmol) who underwent revascularization compared with medically treated patients.^7^ The explanation could be that lower levels of albuminuria indicate better preserved renal parenchyma, making the kidneys more likely to respond to revascularization. In our study, we were unable to use the CORAL albuminuria threshold as albuminuria was extrapolated from proteinuria as described in the methods.^8^ However, in our analysis, there was no statistical difference between outcomes for those with an ACR above or below 30 mg/mmol.

An important limitation, and one that has been well highlighted, was that the ASTRAL population included lower-risk patients (40.8% of patients had RAS <70%) and did not include a large number of high-risk patients, for example, those with severe hypertension or pulmonary edema. As highlighted earlier, both ASTRAL and CORAL excluded patients who may have benefited from revascularization based upon clinician choice, and these were then managed outside of the trials, questioning the real world applicability of the RCTs to the management of more severely affected RAS patients. During the course of the study, 13% of patients withdrew their consent or were lost to follow-up. Although this proportion is quite high, it is recognized that the study was conducted over 13 years (from 2000 to 2013) in atherosclerotic renovascular disease patients across 53 separate UK and 4 Australasian centers. Analyses show that patients lost to follow-up were similar in both trial arms and did not affect the study outcomes (Table 1; Table S1).

The overall rate of renal events and RRT was low after entry into ASTRAL, implying that in the majority of patients, atherosclerotic renovascular disease does not lead to progressive loss of kidney function once vasculo-protective medications are initiated. There was a high occurrence of CVEs, and annual mortality was 4.7% in the medical group and 4.6% in the intervention arm during the 12 years of follow-up in this RCT. These annual event rates were lower overall than during the original follow-up period, and this is likely to reflect both survivor bias and improved cardiovascular outcomes over time in these high-risk patients, the latter highlighting the importance of preventative medical therapies.^9^

Hence, despite the results of these RCTs, there remains uncertainty about management. Many clinicians continue to adopt an individualized approach, taking into consideration the potential viability of the kidney (size, proteinuria, and renal function), the degree of RAS (>70% and key computed tomography or magnetic resonance angiography features), and emphasis is now placed on the clinical presentation (severe hypertension, heart failure, and deteriorating renal function) of the patient.^10^ There are case series and other non-RCT data that support intervention in these populations.^11–15^ There remains equipoise about intervention in this subset of patients, which may be worthy of further study in a more selected population as recommended in the recent Kidney Disease Improving Global Outcomes controversy paper on this topic.^10^ The American Heart Association has also published a recent statement on the benefits of selective revascularization and where this may be considered.^16^ In practice, recruitment for a trial in this cohort may be difficult given that patients may be acutely unwell (eg, pulmonary edema) or clinicians may be unwilling for their patients to potentially be randomized to no intervention where they perceive a benefit; this was noted in RADAR, which terminated prematurely partly because of slow recruitment due to investigator apathy after publication of ASTRAL.^17^

In conclusion, long-term follow-up of the ASTRAL trial population found no significant difference between endovascular revascularization plus medical therapy compared with medical therapy alone in a population that overall had modest atherosclerotic RAS.

## ARTICLE INFORMATION

### Acknowledgments

The authors wish to acknowledge the contribution of Natalie Ives, Senior statistician at the Birmingham Clinical Trials Unit, to the original study.

### Sources of Funding

The initial ASTRAL study was supported by research grants from Medical Research Council UK, Kidney Research UK, and Medtronic. The University of Birmingham Trials Unit was also receiving core support from the UK Department of Health.

### Disclosures

Dr Green has received speaker fees or consultancy fees from AstraZeneca, Bayer, GlaxoSmithKline, Novartis, Boehringer-Ingelheim, and Lilly. The other authors report no conflicts.

### Supplemental Material

Table S1

Figure S1

List of ASTRAL Investigators and Centers

## Supplementary Material


