# Supplementary material for: Long Term Outcomes After Renal Revascularization for Atherosclerotic Renovascular Disease in the ASTRAL Trial
Source: Circ Cardiovasc Interv. 2024 Aug 15;17(9):e013979. doi: 10.1161/CIRCINTERVENTIONS.123.013979 (PMC11404757; doi:10.1161/CIRCINTERVENTIONS.123.013979)
Supplement: Supplementary file 1 [file hcv-17-e013979-s001.pdf]

SUPPLEMENTAL MATERIAL

Supplementary Results:

Figure S1: Per protocol analysis of Blood Pressure over time (1=revascularized, 2=not revascularized, 3=medical arm).

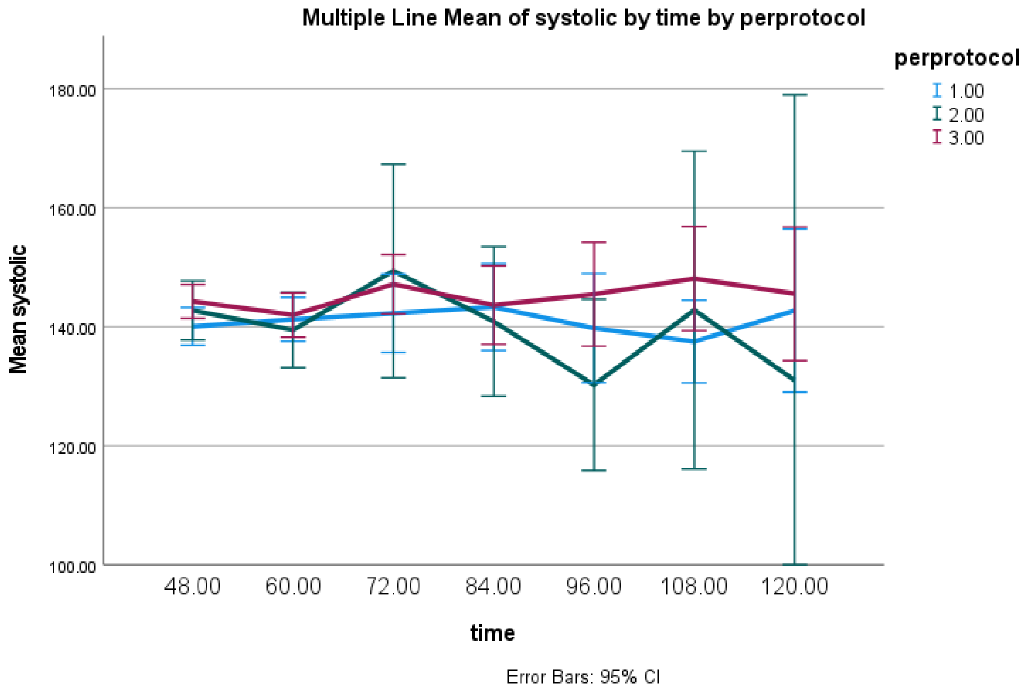

Table S1: Cumulative incidence probability for outcomes (death, renal event, cardiovascular event and lost to follow-up) between the groups (medical vs revascularization) in a competing risk analysis

Months from study entry	Groups	Death	Renal event	Cardiovascular event	Lost to follow up
20	Medical	0.065	0.015	0.219	0.015
	Revascularization	0.055	0.030	0.240	0.035
40	Medical	0.117	0.048	0.312	0.028
	Revascularization	0.106	0.047	0.296	0.035
60	Medical	0.164	0.056	0.374	0.050
	Revascularization	0.162	0.053	0.347	0.045
80	Medical	0.222	0.060	0.412	0.071
	Revascularization	0.213	0.060	0.373	0.060
100	Medical	0.242	0.060	0.437	0.091
	Revascularization	0.254	0.064	0.393	0.060
	**p-Value**	0.623	0.895	0.348	0.587

ASTRAL Investigators and Centers

Data Monitoring Committee:

N. Buller, M. Flather (Chair), P. Royston, P. Whelton.

Serious Adverse Event Safety Committee:

N. Ives, PA. Kalra, JG. Moss.

Trial Management Committee:

C. Baigent, S. Carr, N. Chalmers, D. Eadington, K. Fitzpatrick-Ellis, R. Gray, G. Hamilton, N. Ives, PA. Kalra (nephrology lead), G. Lipkin, JG. Moss (radiology lead), A. Nicholson, J. Scoble, K. Wheatley.

Trial Steering Committee:

D. Clayton, D. Crane, A. Dixon, PA. Kalra (nephrology lead), N. Mallick (Chair), JG. Moss (radiology lead), M. Roberts (MRC observer), J. Voight (MRC observer), K. Wheatley.

University of Birmingham Clinical Trials Unit (trial co-ordination, IT and analysis):

E. Adey, J. Daniels, K. Fitzpatrick-Ellis, R. Gair, S. Goodsell, K. Handley, J. Harding, N. Hilken, N. Ives, S. Jeyaseelan, M. Nixon, L. Patel, S. Patel, L. Scott, M. Wilks.

The following centers and investigators (listed in alphabetical order) participated in the trial: (figures in parentheses indicate number of patients entered) (the Principal Investigator at each center is indicated by *):

Aberdeen Royal Infirmary (32) - W. Crichton, J. Furnace, P. Thorpe, J. Webster*, K. Witte;

Antrim Area Hospital, Northern Ireland (2) – C. Harron*;

Arrowe Park Hospital, Wirral (7) – R. Klenka, M. Lipton, J. Magennis, P. McClelland*;

Ayr & Crosshouse Hospitals (2) – A. Innes, P. MacKenzie, I. McKaye*;

Belfast City Hospital, Northern Ireland (3) – H. Brown*, C. Doherty, D. Fogarty, R. Hannon, G. Johnston, L. Johnston, W. Loan, P. Maxwell;

Birmingham Heartlands Hospital (14) – M. Carmoody, PM. Crowe, J. Henderson, SA. Smith, M. Temple, M. Thomas*;

Charing Cross Hospital, London (2) – E. Brown*, A. Frankel, RM. Greenhalgh, J. McIvor, A. Mitchell, M. Roddie;

Christchurch Hospital, New Zealand (4) – A. Laing, D. McGregor, M. Searle*, J. Usher;

Conquest Hospital, St Leonards-on-Sea (1) – JA. Giles*;

Countess of Chester Hospital (3) – G. Abbott, J. Clements, A. Crowe*, P. Fitzgerald, M. Johnson, G. King, W. McMahon, GR. Sissons;

Derby City General Hospital (10) – M. De Nunzio, R. Fluck*, CW. McIntyre;

Derriford Hospital, Plymouth (38) – H. Cramp, W. Douie, RJ. McGonigle, CA. Roobottom*, P. Rowe, W. Tse, I. Wells, C. West;

Dorset County Hospital (providing patient follow-up) – J. Taylor*;

Dumfries and Galloway Hospital (7) – A. Brammah, J. Dreyer, D. Hill, C. Isles*, K. Isles, S. Robertson, G. Watson;

Edinburgh Royal Infirmary (5) – P. Gibson, I. Gillespie*, J. Goddard, S. Ingram, C. Whitworth, R. Winney;

Freeman Hospital, Newcastle-upon-Tyne (4) – M. Ablet, A. Brown*, T. Chaudry, R. Christer, P. Haslam, R. Jackson, S. Kanagasundaran, HWC. Loose, S. MacDonald, L. Mitchell, L. Murthy, R. Owen, J. Rose, JS. Tapson, MK. Ward;

Frimley Park & St Helier Hospitals (16) – P. Andrews, M. Bending, K. Bundy, A. Eisinger, JRW. Hall, F. Harris, A. Hatrick*, A. Keane, A. Keightley, J. Kwan, JE. Marsh, H. Massouh, T. North, N. Velasco;

Gartnavel Hospital & Western Infirmary, Glasgow (46) – D. Briggs, JM. Connell, C. Daly, AF. Dominiczak, R. Edwards, C. Geddes, A. Gordon, M. Gorrie, J. Innes, A. Jardine, BJ. Junor, M. MacDonald, E. McGregor, M. McIntyre, F. McLean, M. McMillan, PA. Meredith, JJ. Morton, JG. Moss*, N. Padmanabhan, I. Robertson, S. Rodger, P. Semple;

Glan Clwyd Hospital (16) – S. Baikunje, R. Byrne, R. Glover, MJ. Kumwenda*, C. McConnell, E. Moss;

Glasgow Royal Infirmary (23) – C. Deighan*, C. Ferguson, J. Fox, R. MacTier, G. Roditi, K. Simpson;

Gloucester Royal Hospital (3) – R. Banks*, P. Birch, A. Williams;

Gosford Hospital, Australia (21) – E. Bohringer, L. Brady, S. Roger*;

Guy's Hospital, London (28) – M. Anodu, D. Goldsmith, S. Heffernan, J. Reidy, S. Sacks, J. Scoble*, S. Thomas, J. Watkins;

Gwynedd District Hospital (3) – T. Bedson, P. Birch*, L. Bloodworth, H. Godfrey, M. Jibani, J. Owen, MK. Phanish;

Hairmyres Hospital, East Kilbride (10) – M. Hand, S. Millar*, G. Moreland, J. Young;

Hull Royal Infirmary (26) – K. Bel'Eed, J. Cleland, J. Dyet, D. Eadington*, D. Ettles, MJ. Farr, P. Huan Loh, D. Lewis, PT. McCollum, G. Robinson, P. Scott, L. Sellars;

Ipswich Hospital (2) – G. Glancey, G. Picken*, P. Whitear, P. Williams;

Launceston General Hospital, Tasmania (34) – L. Anderson, D. Cooke, R. Fassett, J-S. Gan, B. Herman, MC. Mathew*, M. Smith;

Leicester General Hospital (30) – J. Barratt, K. Blanshard, A. Bolia, N. Brunskill, S. Carr*, S. Dickinson, H. El-Shazly, J. Feehally, B. Fentum, G. Fishwick, K. Harris, J. James, K. Krarup, T. Kumar, AJP. Lewington, J. Medcalfe, S. Nicholson, Y. Rees, H. Thurston, P. Topham, M. Turner, G. Warwick, B. Williams;

Lister Hospital, Stevenage (1) – K. Farrington*, A. Fry, C. King, C. Prendergast, P. Warwicker;

Manchester Royal Infirmary (28) – H. Andrew, F. Ballardie, J. Bendle, N. Chalmers*, D. Comer, JK. Cruikshank, K. Czapla, P Durrington, R. Gokal, A. Heagerty, P. Hodson, L. Howard, A. Hutchison, R. Malik, N. Mallick, M. Picton, F. Qasim, CD. Short, JV. Smyth, D. Tunbridge, J. Wright;

Morriston Hospital, Swansea (2) – SE. Evans, T. Davies, D. Roberts, A. Williams*;

Ninewells Hospital, Dundee (35) – S. Chakraverty, N. Gourlay, I. Henderson, JG. Houston*, T. McDonald, AD. Struthers, I. Zealley;

Northern General Hospital, Sheffield (2) – CB. Brown, P. Brown, S. Brown, T. Cleveland, C. Davies, A. El Nahas, H. Euinton, I. Ihmoda, YC. Kuan*, P. Moorhead, A. Procter, S. Thomas, D. Throssell;

Nottingham City Hospital (4) – M. Cassidy, A. Manhire, SD. Roe*;

Pennine Acute Hospital, Rochdale (1) – I. Erekosima, R. Raja, DJ. Smithard*;

Queen Alexandra Hospital, Portsmouth (15) – M. Arkanath, J. Atchley, S. Baikunte, L. Coni, I. Fairley, AF. Hughes, J. Langham Brown, TD. Leach, RJ. Lewis, JC. Mason, JM. Stevens, G. Venkat-Ramen*;

Queen Elizabeth Hospital, Birmingham (51) – D. Adu, S. Ball, G. Beevers, R. Borrows, N. Buller, P. Cockwell, L. Crutch, C. Day, C. Ferro, L. Foggensteiner, V. Gray, H. Green, L. Harper, P. Hewins, K. Horton, H. Ibrahim, H. Joy, C. Kennedy, G. Lipkin*, M. Little, U. Martin, I. McCafferty, F. McGlynn, K. Moniem, S. Olliff, N. Richards, P. Riley, C. Savage, D. Wheeler;

Queen Margaret Hospital, Dunfermline (6) – KS. Buck, T. Daniel, H. Ireland, D. Jenkins, DM. Lewis, K. McBride*, SM. Wood;

Royal Berkshire Hospital, Reading (10) – LC. Barker, M. Gibson, J. King, RB. Naik*;

Royal Cornwall Hospital (6) – J. Barnes, C. Ferris, J. Hancock, P. Johnston*, R. Parry, B. Smith, S. Travis;

Royal Devon and Exeter Hospital (12) – R. D'Souza*, D. Harrison, D. Kinsella, T. Watkinson;

Royal Free Hospital, London (61) – D. Baker, JM. Cross*, A. Davenport, A. Goode, G. Hamilton, R. Kinyanjui, F. Myint, AD. Platts, S. Powis, S. Smith, P. Sweny, J. Tibballs;

Royal Liverpool University Hospital (3) – J. Alexander, A. Bakran, JM. Bone, PS. Carter, E. Garforth, R. McWilliams, M. Mohteshamzadeh*, P. Pai, PC. Rowlands, R. Rustom, P. William;

Royal Perth Hospital, Australia (8) – L. Burnette, A. Irish*;

Royal Preston Hospital (31) – J. Anderton, E. Bailey, RA. Coward, S. D'Souza, SP. Gibson, P. MacDowall, D. Seriki, L. Solomon*;

Royal United Hospital, Bath (6) – R. Bradley, A. Chalmers, J. Fretwell, C. Hall*, J. Hardman, S. Hayward, M. Horrocks, C. John;

Salford Royal Hospital, Salford (74) – C. Cheung, T. Chrysochou, A. Cowie, A. El'Deen Shurrab, S. Gowland, D. Green, L. Haydock, J. Hegarty, PA. Kalra*, H. Mamtora;

Southmead Hospital, Bristol (5) – AJ. Armitage, C. Burton, C. Dudley*, L. Dudley, T. Feest, S. Harper, J. Haworth, P. Lear, E. Loveday, P. Mathieson, D. Mitchell, J. Parkin, SC. Satchell, RM. Smith, M. Thornton, CRV. Tomson;

St George's Hospital, London (4) – A. Belli, C. Lawson, G. MacGregor*; P. Swift;

St James' University Hospital, Leeds (7) – D. Burwell, D. Kessel, A. Mooney*, CG. Newstead, A. Nicholson, J. Patel;

St Luke's Hospital, Bradford (12) – H. Akbani, J. Barber, R. Jeffrey*, R. Lowe;

Walsgrave Hospital, Coventry (2) – N. Aldridge, S. Hewins, R. Higgins, J. Rush, D. Zehnder*;

Wishaw General Hospital (1) – M. Fleet*;

Withington Hospital, Wythenshawe (12) – P. Ackrill, R. Ashleigh*, D. Martin, M. Venning;

Worcestershire Royal Hospital (8) – P. Slaney, S. Spencer*;

York Hospital (3) – A. Bowker, C. Jones, D. Richardson, N. Warnock, D. Worth*;

Ysbyty Maelor, Wrexham (4) – W. Ahmed, S. Argarwal, P. Drew, D. Glover, SW. Robertson*, J. Welham, V. Wyn-Jones.
